# Prognostic Value of Triglyceride Glucose Index in ST-Elevation Myocardial Infarction: A Key Predictor of Mortality and Thrombus Burden

**DOI:** 10.3390/diagnostics14202261

**Published:** 2024-10-11

**Authors:** Murat Bilgin, Emre Akkaya, Recep Dokuyucu

**Affiliations:** 1 Department of Cardiology, Private Aktif International Hospital, Yalova 77720, Turkey; drbilginmurat61@gmail.com; 2 Department of Cardiology, Bossan Hospital, Gaziantep 27580, Turkey; dremreakkaya@hotmail.com; 3 Department of Physiology, Medical Specialization Training Center (TUSMER), Ankara 06230, Turkey

**Keywords:** ST-elevation myocardial infarction, triglyceride glucose index, TIMI thrombosis score, mortality

## Abstract

Objectives: We aimed to investigate the association between the triglyceride glucose index (TGI) and mortality in patients with ST-elevation myocardial infarction (STEMI). Methods: This retrospective study utilized data from the records of patients diagnosed with STEMI who underwent primary percutaneous coronary intervention (PCI) at the Cardiology Department of Private Aktif International Hospital between 2020 and 2023. Demographic data, medical history, laboratory results, and treatment processes of the patients were obtained from retrospective records. Patients were divided into low (TGI ≤ 8.6)-, medium (TGI = 8.6–9.2)-, and high (TGI ≥ 9.2)-TGI groups according to their TGI levels. Results: The average age of the patients was 62 ± 10 years, and 65% were men. The intracoronary thrombus burden of patients in the high-TGI group was found to be significantly higher compared to the low- and medium-TGI groups (*p* = 0.01). While the rate of patients with a thrombolysis in myocardial infarction (TIMI) thrombosis score of 3 or above was 45% in the high-TGI group, this rate was observed to be 20% in the low-TGI group. The short-term (30-day) mortality rate was found to be 15% in the high-TGI group, 8% in the medium-TGI group, and 5% in the low-TGI group (*p* = 0.02). Long-term (after 30 days) mortality rates were determined as 25% in the high-TGI group, 15% in the medium-TGI group, and 10% in the low-TGI group (*p* = 0.01). TGI was an independent risk factor for both short-term and long-term mortality. The hazard ratio (HR) of high TGI levels for short-term mortality was found to be 2.5 (95% CI: 1.5–4.1, *p* = 0.01), and the HR for long-term mortality was 2.0 (95% CI: 1.3–3.2, *p* < 0.02). Conclusions: Our results show that high TGI levels are associated with increased thrombus burden and high mortality rates in STEMI. TGI can be used not only in predicting STEMI but also in early risk stratification and treatment planning for STEMI patients.

## 1. Introduction

Acute myocardial infarction (AMI) is a leading cause of hospitalizations and mortality worldwide, with ST-elevation myocardial infarction (STEMI) being particularly deadly due to complete arterial blockage [1]. STEMI occurs as a result of complete blockage of the heart artery. Despite advancements in reperfusion and medical therapies, STEMI remains a significant cause of cardiovascular deaths. The systemic inflammatory response triggered by acute STEMI is crucial for myocardial repair, and elevated inflammatory markers are linked to severe coronary atherosclerosis and poorer outcomes post-intervention [2]. This pro-inflammatory response involves the release of various cells and molecules into the bloodstream. Studies in the literature have linked altered levels of inflammatory markers to more severe coronary atherosclerosis, acute coronary syndromes, and poorer outcomes after coronary interventions [3,4,5]. Some studies suggest that certain biomarkers can predict mortality in critically ill patients upon admission, indicating their potential utility for risk stratification in STEMI patients [4,5,6]. While biomarkers are increasingly recognized for risk stratification, the specific prognostic value of the triglyceride glucose index (TGI) in STEMI patients is not well established.

The idea that the triglyceride glucose index (TGI) could be used as a parameter indicating insulin resistance has arisen due to the expensiveness of the insulin test and the difficulty of accessing the test in underdeveloped countries [7]. TGI, calculated using fasting blood sugar and triglyceride levels, which are among the routine examinations in clinical practice, is a low-cost and easily calculated parameter. Studies have shown that it is superior to the Homeostasis Model Assessment of Insulin Resistance (HOMA-IR) in predicting insulin resistance [8,9,10]. Several factors contribute to the development of cardiovascular disease, including glycemic abnormalities and lipid imbalances. Hypertriglyceridemia, a common form of dyslipidemia, has a debated relationship with cardiovascular disease risk. Nevertheless, it is considered an independent risk factor for glucose metabolism disorders [11]. Elevated plasma triglyceride levels are strongly linked to high glucose levels due to interactions between fat, muscle, and pancreatic β-cell function [7]. Studies have indicated that high-normal fasting glucose and triglyceride levels can predict cardiovascular disease risk. The TGI has been found to be a more accurate predictor of cardiovascular events than either triglyceride or glucose levels alone [12]. Additionally, TGI is a valuable predictor of type 2 diabetes and metabolic syndrome and contributes to overall cardiometabolic risk. Numerous studies have demonstrated associations between TGI and hypertension, arterial stiffness, and coronary artery calcification [12,13,14].

However, despite the growing body of evidence on TGI in various cardiovascular contexts, its prognostic value specifically in STEMI patients remains insufficiently explored. Current literature suggests that TGI could play a role in identifying patients at higher risk of adverse outcomes, particularly in those undergoing primary percutaneous coronary intervention (PCI), yet definitive conclusions regarding its impact on mortality and long-term prognosis are lacking.

This study aims to address this research gap by evaluating the effect of TGI on both short- and long-term mortality in STEMI patients undergoing primary PCI. By investigating this relationship, we seek to provide further insights into the utility of TGI in clinical practice, potentially enhancing risk stratification and guiding therapeutic decisions for STEMI patients.

## 2. Materials and Methods

### 2.1. Study Design and Study Population

**Figure 1:**
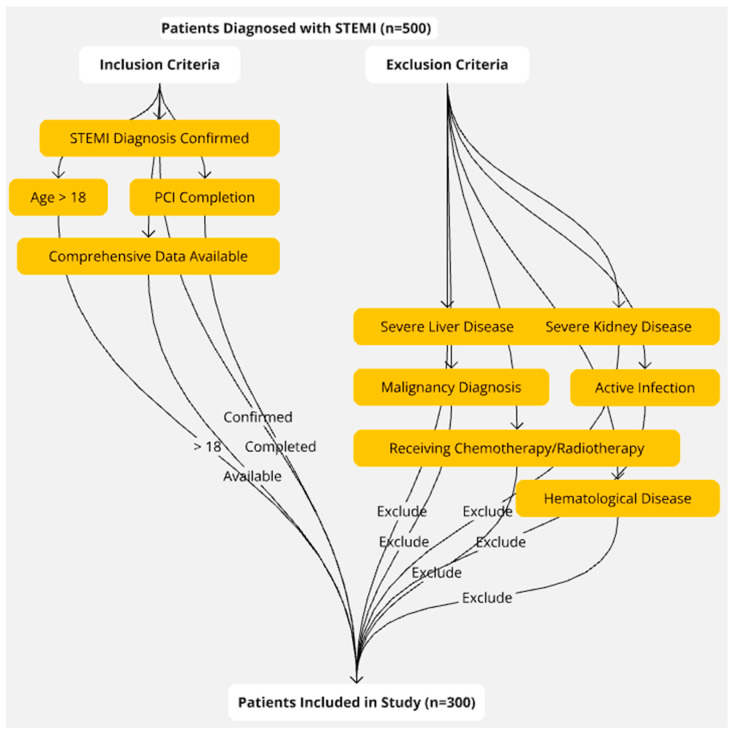
Low-TGI group: TGI ≤ 8.6.

This retrospective study included consecutively enrolled patients diagnosed with ST-elevation myocardial infarction (STEMI) who underwent primary PCI at the Cardiology Department of Private Aktif International Hospital between 2020 and 2023. Inclusion criteria were a confirmed STEMI diagnosis over the age of 18, completion of PCI, and comprehensive data availability. Exclusions from the study included patients diagnosed with malignancy, severe liver or kidney disease, or active infection, receiving chemotherapy/radiotherapy, and those with hematological disease. After exclusion criteria (*n* = 200), a total of 300 patients diagnosed with NSTEMI were included in the study (Figure 1).

The diagnosis of STEMI was made according to the 2023 ESC Guidelines for the management of acute coronary syndromes. The diagnosis was based on clinical symptoms, ECG findings, elevated levels of cardiac biomarkers, such as troponin, and on the ST-elevation on the ECG [15].

Intermediate-TGI group: TGI between 8.6 and 9.2.

High-TGI group: TGI ≥ 9.2 [16,17].

Triglyceride glucose index is calculated using the formula:

TGI = ln(triglyceride [mg/dL] × glucose [mg/dL]/2).

The triglyceride glucose index (TGI) was calculated using blood samples taken upon admission to the hospital. This timing was chosen to reflect the acute metabolic status of the patients at the onset of ST-elevation myocardial infarction (STEMI) and prior to any therapeutic interventions that might affect glucose or triglyceride levels. No additional TGI measurements were taken at 24 h or discharge.

### 2.2. Intracoronary Thrombosis Evaluation

Intracoronary thrombus burden was assessed in all patients using angiographic images obtained during PCI. The thrombus burden was graded according to the thrombolysis in myocardial infarction (TIMI) thrombus grading system, which classifies thrombus burden into six categories based on angiographic characteristics:

Grade 0: No evidence of thrombus.
Grade 1: Possible thrombus is present, with reduced contrast density or haziness, but without visible thrombus.
Grade 2: Small thrombus, with the greatest dimension less than half the vessel diameter.
Grade 3: Moderate thrombus, greater than half but less than twice the vessel diameter.
Grade 4: Large thrombus, with the greatest dimension twice or more than the vessel diameter.
Grade 5: Complete occlusion of the vessel due to thrombus.

High thrombosis burden was defined as a TIMI score of 3 or above [18], indicating the presence of significant thrombus and a higher risk of adverse clinical outcomes.

### 2.3. Mortality Tracking

Mortality data of the patients were tracked using hospital records and the national death registration system. Short-term mortality was defined as deaths within the first 30 days, and long-term mortality included deaths after 30 days, with follow-up of 1 year. The primary endpoint of the study was all-cause mortality. Mortality was analyzed for all causes, not restricted to cardiovascular-related events. This approach allowed us to capture a broader range of potential outcomes associated with TGI levels.

### 2.4. Statistical Analysis

Data analysis was performed using SPSS (Statistical Package for the Social Sciences) version 27.0. Continuous variables are expressed as mean ± standard deviation, and categorical variables are presented as frequencies and percentages. Before conducting parametric tests, the normality of continuous data distributions was assessed using the Shapiro–Wilk test. For comparisons between groups, Student’s *t*-test was used for normally distributed continuous variables, while the Mann–Whitney U test was applied for non-normally distributed variables. Categorical data were analyzed using the Chi-square test or Fisher’s exact test when the expected frequencies were low. For the multivariate Cox proportional hazards regression analysis, covariates were selected based on clinical relevance and previous literature [19,20,21,22,23]. The proportionality assumption was checked using Schoenfeld residuals. To avoid model overfitting, we adhered to the rule of including no more than one covariate per 10 outcome events. Additionally, multicollinearity between variables was assessed using the variance inflation factor (VIF), and covariates with a VIF > 5 were excluded from the model to minimize the risk of multicollinearity. Statistical significance was defined as *p* < 0.05.

## 3. Results

A total of 300 patients with ST-elevation myocardial infarction (STEMI) were included in the study. The average age of the patients was 62 ± 10 years, and 65% were men. Patients were divided into low-, medium-, and high-TGI groups according to their TGI levels. Demographic and clinical characteristics are presented in Table 1. Patients in the high-TGI group tend to be older (64 ± 12 years) compared to the medium- (62 ± 11 years) and low-TGI (60 ± 10 years) groups, with the total average age being 62 ± 11 years. The prevalence of hypertension, diabetes mellitus, and smoking increase with higher TGI levels compared to the medium and low group. The LAD is the most commonly involved artery, with involvement increasing from 35% in the low-TGI group to 45% in the high-TGI group. Peri-procedural complications were observed across the three TGI groups, and included various adverse events related to the PCI procedure. In the low-TGI group, 5% (5/100) of patients experienced complications, while 8% (8/100) and 12% (12/100) of patients in the medium- and high-TGI groups, respectively, had complications. These included bleeding complications, access site complications, coronary artery dissection, no-reflow phenomenon, and arrhythmias. Left ventricular ejection fraction (LVEF) decreases as TGI increases, with the high-TGI group having the lowest LVEF (45 ± 10%) compared to the low group (55 ± 7%). The mean TGI increased significantly across the groups, from 5.2 ± 0.5 in the low group to 10.1 ± 0.7 in the high group, showing a clear categorization based on this index (Table 1). 

### 3.1. Triglyceride Glucose Index and Intracoronary Thrombosis Burden

**Figure 2:**
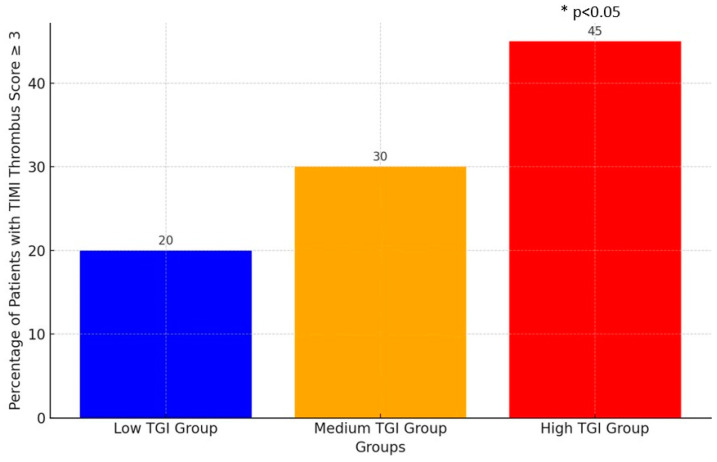
Percentage of patients with high TIMI thrombus score in different TGI groups. TGI: triglyceride glucose index.

The intracoronary thrombus burden of patients in the high-TGI group was found to be significantly higher compared to the low- and medium-TGI groups (*p* = 0.01). While the rate of patients with a TIMI thrombosis score of 3 or above was 45% in the high-TGI group, this rate was observed to be 20% in the low-TGI group (Figure 2).

### 3.2. Mortality Analysis

The short-term (30-day) mortality rate was found to be 15% in the high-TGI group, 8% in the medium-TGI group, and 5% in the low-TGI group (*p* = 0.02). Long-term (after 30 days) mortality rates were determined as 25% in the high-TGI group, 15% in the medium-TGI group and 10% in the low-TGI group (*p* = 0.01) (Table 2). The median follow-up period for long-term mortality was 3.5 years (interquartile range: 1.6–5.2 years).

### 3.3. Cox Regression Analysis

Cox regression analysis showed that TGI was an independent risk factor for both short-term and long-term mortality. The hazard ratio (HR) of high TGI levels for short-term mortality was found to be 2.5 (95% CI: 1.5–4.1, *p* = 0.01), and the HR for long-term mortality was 2.0 (95% CI: 1.3–3.2, *p* = 0.02) (Table 3).

## 4. Discussion

This study examined the effect of TGI on intracoronary thrombus burden and mortality in patients with STEMI. The results of our study show that higher TGI levels are significantly associated with increased intracoronary thrombus burden and mortality rates. Therefore, it strengthens the possibility of using TGI as a marker in predicting STEMI.

Studies in the literature have reported a relationship between TGI and risk factors in cardiovascular diseases and metabolic diseases. In their study, Liu et al. reported a relationship between the risk of developing hypertension and TGI [23]. In the study conducted by Zhang et al., it was reported that there is a relationship between TGI levels and the risk of developing hypertension, and that high TGI values are associated with the risk of developing hypertension [24]. A study by Cho et al. reported a strong association between high TGI levels and insulin resistance and atherosclerotic cardiovascular diseases [19]. In a study by Silva et al., the association between TGI and symptomatic CAD in patients receiving secondary care was examined [20]. The study focused on patients who had experienced at least one cardiovascular disease (CVD) event within the past decade. Participants were categorized into three groups: asymptomatic individuals, those with symptoms, and those undergoing treatment for CAD. Upon calculating the TGI across these groups, a statistically significant difference emerged specifically within the symptomatic group [20]. The findings indicated that a higher TGI was more prevalent among symptomatic patients. This association remained significant even after conducting regression analyses on all groups, accounting for variables such as sex, age, and the use of hypoglycemic, antihypertensive, anticoagulant, and lipid-lowering medications [20]. In the study conducted by Jin et al., it was reported that there was a positive correlation between TGI and the risk of recurrent cardiovascular events in ACS patients, and that TGI had a high prognostic value [22]. A study by Wang et al. reported that TGI increased the risk of major adverse cardiovascular events (MACE) in patients with stable CVD, and that clinical outcomes in CVD patients worsened as TGI levels increased [25]. In our study, in addition to findings in the literature, we found that TGI in STEMI patients is associated with increased intracoronary thrombus burden and poor clinical outcomes. In terms of potential mechanisms, elevated TGI levels may trigger oxidative stress and inflammation, accelerating atherosclerosis and worsening cardiac damage. Disruptions in lipid and glucose metabolism contribute to vascular inflammation and microvascular dysfunction, leading to poorer clinical outcomes. These mechanisms highlight the prognostic significance of TGI in STEMI patients, and further studies exploring these relationships in detail would be beneficial.

A limited number of studies have reported that TGI is an effective marker in predicting cardiovascular disease risk and prognosis [21,26,27,28]. The significant relationship between high TGI levels and intracoronary thrombus burden and mortality, especially in STEMI patients, suggests that TGI can be used as a prognostic marker in clinical practice [29]. This possibility suggests that TGI may have an important role not only in cardiovascular risk factors but also in the treatment management of STEMI patients. A meta-analysis study reported a strong association between TGI and cardiovascular disease and mortality. It has also been reported that high TGI levels are associated with cardiovascular events and deaths in the general population [30]. Another study reported that TGI increases the risk of MACE in patients with stable CAD and that HbA1c and TGI when used together can be used to predict prognosis [9]. It has also been shown that high TGI levels increase the likelihood of coronary artery stenosis and plaque formation [31]. In the study by Luo et al., it was found that high TGI was associated with poor prognosis in patients with acute STEMI after PCI. STEMI patients who underwent PCI were included in the study [32]. Patients were categorized into four distinct groups based on their TGI levels. Clinical characteristics, fasting plasma glucose, triglycerides, various other biochemical markers, and the occurrence of major adverse cardiovascular events (MACE) were monitored throughout the follow-up period. It was observed that the incidence of MACEs and all-cause mortality at 30 days, 6 months, and 1 year post-PCI was significantly elevated among STEMI patients with the highest TGI levels [32]. In STEMI patients, within 1 year after PCI, a 1.529-fold increased risk of MACE was found for those in the highest-TGI group, regardless of confounding factors. The area under the curve of the TGI, which predicts MACE formation after PCI in STEMI patients, was found to be 0.685. This study found that higher TGI levels in STEMI patients were associated with an increased risk of MACE, and it was stated that TGI may be a valid predictor of clinical outcomes in STEMI patients undergoing PCI [32]. Karadeniz et al. investigated the prognostic value of the TGI in patients with ACS. The findings indicate that a higher TGI (≥8.65) is significantly associated with an increased risk of MACEs during both in-hospital and long-term follow-up (60 months). It was stated that the TGI is a potential predictor of poor outcomes in ACS patients [33]. Pang et al. explored the relationship between the TGI, its components, and the incidence of cardiovascular events in patients with ACS. It was suggested that a higher TGI and elevated levels of its components are independently associated with an increased risk of cardiovascular events, including mortality, during follow-up. This emphasizes the potential utility of the TGI as a predictive marker in clinical practice [34]. Li et al. investigated the role of the TGI as a predictor of cardiovascular outcomes in patients with NSTEMI. They stated that a higher TGI is significantly associated with worse cardiovascular outcomes, including increased risk of mortality and adverse events during follow-up. It was suggested that the TGI could serve as a valuable tool for risk stratification in NSTEMI patients [35]. In our study, we found that increased thrombus burden and mortality risk in STEMI patients was associated with high TGI levels. Our study is one of a limited number of studies examining the effects of TGI on intracoronary thrombus burden and mortality in STEMI patients. Findings from previous studies support that TGI is an important marker for predicting cardiovascular disease risk and prognosis. However, our study makes a significant contribution to the literature by revealing the prognostic value of TGI, especially in STEMI patients. These findings suggest that TGI can be used not only in cardiovascular risk factors but also in the management of STEMI patients.

In addition to laboratory markers like TGI, the role of imaging techniques such as cardiac magnetic resonance (CMR) has become increasingly valuable in the prognostic stratification of STEMI patients. CMR is particularly useful in assessing inflammation and fibrosis both in the infarcted and remote myocardium, which are crucial for evaluating long-term outcomes in STEMI. Studies have shown that myocardial edema and fibrosis, visualized using late gadolinium enhancement (LGE) and T2-weighted imaging, offer significant prognostic information regarding patient recovery and the likelihood of adverse cardiovascular events [36,37]. Moreover, it is essential to recognize that patients with myocardial infarction with nonobstructive coronary arteries (MINOCA), including those with MINOCA-STEMI, are also at high risk for adverse outcomes. Monitoring metabolic parameters such as TGI and LDL in these patients is critical for risk stratification and reducing the likelihood of re-infarction during follow-up [38].

### Limitations of the Study

Our study has several limitations. First, the retrospective design may introduce selection bias, despite our careful review of patient files. Additionally, the single-center nature of the study and the relatively small sample size limit the generalizability of our results. Therefore, larger multi-center studies are needed to confirm the prognostic value of TGI in STEMI patients. Moreover, while our findings suggest a significant association between TGI and mortality, future research should focus on integrating TGI with other clinical and imaging parameters to improve prognostic accuracy. While TGI shows promise as a valuable tool in clinical applications, its use may be limited by factors such as variability in cut-off values across different populations and potential confounding effects from underlying metabolic conditions, which warrant further investigation. Given these limitations, the results should be interpreted cautiously, and further validation in larger, prospective cohorts is essential.

## 5. Conclusions

In conclusion, our study demonstrates a significant association between elevated TGI levels and increased thrombus burden and mortality in STEMI patients undergoing primary PCI. These findings suggest that TGI could serve as an important cardiovascular risk marker, with potential utility in both early risk stratification and treatment planning. Given the strong relationship of TGI with insulin resistance, metabolic syndrome, obesity, and cardiovascular disease, we propose that TGI could be a valuable and accessible tool in routine clinical practice.

One of the key contributions of our research is its implication for patient management, as patients with higher TGI levels may require more aggressive therapeutic interventions and close monitoring due to their increased risk of thrombus burden and mortality. This could enhance decision-making in the acute setting and improve long-term patient outcomes. Moreover, the ease of measuring and calculating TGI supports its integration into clinical workflows.

Future research should aim to validate these findings through prospective studies and clinical trials, and explore the incorporation of TGI into existing guidelines for STEMI management. Further studies will also clarify the broader prognostic utility of TGI in cardiovascular care, ensuring that it becomes a reliable marker in guiding personalized treatment strategies.

## Figures and Tables

**Table 1:** Demographic and clinical characteristics.

Characteristic	Low-TGI Group (*n* = 100)	Medium-TGI Group (*n* = 100)	High-TGI Group (*n* = 100)	Total (*n* = 300)
Age (Years)	60 ± 10	62 ± 11	64 ± 12	62 ± 11
Gender (Male)	65% (65/100)	70% (70/100)	60% (60/100)	65% (195/300)
Hypertension (%)	30% (30/100)	35% (35/100)	40% (40/100)	35% (105/300)
Diabetes Mellitus (%)	20% (20/100)	25% (25/100)	30% (30/100)	25% (75/300)
Smoking (%)	25% (25/100)	30% (30/100)	35% (35/100)	30% (90/300)
Number of Arteries Involved	1.5 ± 0.6	2.0 ± 0.8	2.3 ± 0.9	1.9 ± 0.7
- LAD (%)	35% (35/100)	40% (40/100)	45% (45/100)	40% (120/300)
- LCx (%)	20% (20/100)	25% (25/100)	30% (30/100)	25% (75/300)
- RCA (%)	30% (30/100)	35% (35/100)	40% (40/100)	35% (105/300)
- Left Main (%)	5% (5/100)	10% (10/100)	15% (15/100)	10% (30/300)
Use of Thrombolysis (%)	20% (20/100)	25% (25/100)	35% (35/100)	27% (80/300)
Peri-procedural Complications (%)	5% (5/100)	8% (8/100)	12% (12/100)	8% (25/300)
LVEF (%)	55 ± 7	50 ± 8	45 ± 10	50 ± 8
Kidney Function (Creatinine, mg/dL)	1.0 ± 0.2	1.2 ± 0.3	1.4 ± 0.4	1.2 ± 0.3
TGI (Mean ± SD)	5.2 ± 0.5	8.8 ± 0.6	10.1 ± 0.7	8.0 ± 0.6

TGI: triglyceride glucose index.

**Table 2:** Mortality analysis with *p*-values and numbers.

Characteristic	Low-TGI Group (*n* = 100)	Medium-TGI Group (*n* = 100)	High-TGI Group (*n* = 100)	*p*-Value
Short-term Mortality (30 days)	5% (5/100)	8% (8/100)	15% (15/100)	0.02
Long-term Mortality (after 30 days)	10% (10/100)	15% (15/100)	25% (25/100)	0.01

TGI: triglyceride glucose index.

**Table 3:** Cox regression analysis.

Mortality	Hazard Ratio (HR)	95% Confidence Interval (CI)	*p*-Value
Short-term Mortality (30 days)	2.5	1.5–4.1	0.01
Long-term Mortality (after 30 days)	2.0	1.3–3.2	0.02
Covariates (Univariate Analysis)			
Age	1.2	1.1–1.5	0.03
Gender	1.1	0.9–1.3	0.12
Hypertension	1.3	1.0–1.6	0.04
Diabetes Mellitus	1.4	1.1–1.7	0.05
Smoking	1.2	0.8–1.6	0.09
TGI	2.5	1.8–3.5	<0.01

## Data Availability

The original contributions presented in the study are included in the article, further inquiries can be directed to the corresponding authors.
